# Trait- versus state- grey matter volume alterations in premenstrual dysphoric disorder

**DOI:** 10.1186/s12888-025-07533-5

**Published:** 2025-12-02

**Authors:** Louise Stiernman, Manon Dubol, Inger Sundström-Poromaa, Marie Bixo, Erika Comasco

**Affiliations:** 1 Department of Clinical Sciences, Umeå University, Umeå, 901 87 Sweden; 2 Department of Women’s and Children’s Health, Science for Life Laboratory, Uppsala University, Uppsala, 752 37 Sweden; 3 Department of Women’s and Children’s Health, Uppsala University, Uppsala, 752 37 Sweden

**Keywords:** Premenstrual dysphoric disorder, Voxel-based morphometry, Grey matter, Menstrual cycle, Mental health

## Abstract

**Background:**

Premenstrual dysphoric disorder (PMDD) is characterized by symptoms of irritability, affective lability, anxiety, and depression, which occur only in the luteal phase of ovulatory menstrual cycles. This offers an ideal model to assess the neural correlates of the on and off switch of mood symptoms. Recently, we highlighted differences in grey matter volume between individuals with PMDD and healthy controls during the luteal phase, depicting smaller volumes in those diagnosed with the condition. However, it is unknown whether such alterations represent state-like changes specific to the symptomatic phase, or trait-like characteristics.

**Methods:**

Here, 28 patients with PMDD and 26 controls underwent anatomical magnetic resonance imaging during the mid-follicular and the late-luteal phases of the menstrual cycle. For each time point, we assessed grey matter volumes over the whole brain using voxel-based morphometry.

**Results:**

We found no group-by-phase interaction effect on grey matter volumes, but a main effect of group across menstrual cycle phases, suggesting trait rather than state structural markers of PMDD. Patients displayed smaller volumes compared to controls, primarily in the cerebellum and cuneus, and at a trend-level in ventral occipito-temporal, parietal, paracentral and orbitofrontal areas, as well as the putamen (Cohen’s d range: 0.4–1.1).

**Conclusions:**

These findings suggest that the differences in grey matter volumes found in PMDD are stable across the menstrual cycle and could represent trait-like, vulnerability markers of PMDD.

**Supplementary information:**

The online version contains supplementary material available at 10.1186/s12888-025-07533-5.

## Background

Premenstrual dysphoric disorder (PMDD) is a hormone-related mood disorder characterized by cyclic affective and physical symptoms that peak during the luteal phase of the menstrual cycle, when progesterone and estradiol levels rise and subsequently fall, and subside in the early follicular phase, when the levels of both hormones are low) [1]. The prevalence, illness burden and morbidity of PMDD [2, 3], along with the lack of universally efficient treatment [4], highlight the need for a better understanding of the etiology of this disorder [5].

The neural underpinnings of PMDD are poorly understood [6]. While most neuroimaging studies of PMDD have investigated functional alterations across the menstrual cycle, few studies have examined brain morphological differences between individuals with PMDD and asymptomatic controls. Four early studies of grey matter volumes (GMV) yielded conflicting results, whereby greater GMV [7], mixed findings of both greater and lesser GMV [8], as well as no differences in GMV [9, 10] were reported in individuals with PMDD compared with controls. Previous studies were hampered by small sample sizes, uncertainties regarding scan-timing during the menstrual cycle, and failure to control for important confounding factors such as age, total intracranial volume (TIV), and body mass index (BMI). On the other hand, the largest neuroimaging study of PMDD to date provided robust evidence of smaller GMV in the lingual, fusiform, inferior occipital and cerebellar areas, as well as subcortical regions such as the amygdala and putamen, compared to controls, with a 70% classification accuracy distinguishing patients from controls based on GMV [11]. These results are in line with findings of smaller GMV in other mood disorders sharing similar symptoms as PMDD, including anxiety and depressive disorders [12, 13], and all regions, except the fusiform gyrus and putamen, have been shown to differ between individuals with PMDD and controls in task-based functional neuroimaging studies of PMDD [14]. Moreover, recent reports have shown associations between grey matter structure and PMDD symptoms during the symptomatic luteal phase, including a negative correlation between the volume of the amygdala and the severity of depressive symptoms [15]. Thus, we hypothesized that grey matter abnormalities in PMDD underlie a developmental neurobiological vulnerability which predisposes menstruating individuals to develop the condition, or alternatively reflect neural adaptation to emotional brain networks dysfunction. Nevertheless, the temporal framework of such adaptative mechanisms across the female development is currently unknown.

One major outstanding question is whether grey matter abnormalities reflect trait disorder-specific features of PMDD or state properties tied to the variations in levels of ovarian hormones (estradiol, progesterone) and premenstrual symptoms. Indeed, ovarian hormone fluctuations across the menstrual cycle have been shown to impact structural brain measures in a number of corticolimbic brain regions in healthy, naturally cycling individuals [16, 17], thus providing a framework for potential phase-specific alterations in PMDD. Intriguingly, neuroimaging studies have highlighted alterations in brain function, metabolism and neurotransmission in the brain of individuals with PMDD, which seem to be both dependent (state-like) and independent (trait-like) of current hormonal status [14]. In sum, while there is substantial evidence that individuals with PMDD present an altered sensitivity to hormonal steroid fluctuations [18], some findings suggest that certain differences in brain function are independent of short-term changes in ovarian steroid levels. Whether similar observations can be made at the structural level remains to be investigated.

The present study sought to investigate GMV in individuals with PMDD and controls across the menstrual cycle to determine whether alterations in grey matter structure represent state- or trait characteristics of PMDD. We expected to find differential GMV in areas previously reported to differ between groups in the luteal phase [11].

## Methods

### Participants

The study was carried out at the Umeå University Centre for Functional Brain Imaging, Sweden. A priori power calculations made using G*Power version 3.1.9.7 [19] indicated that, assuming a power of 95%, an α error probability of 0.05 and medium effect sizes (Cohen’s f ≥ 0.25, as defined by Cohen [20]), a total sample size of 54 participants was required to reliably detect significant interaction effects using a repeated measures ANOVA. In total, thirty-two individuals with PMDD and thirty-two healthy controls were recruited by advertisement in local newspapers, a student website for clinical trials, social media platforms, and boards at out-patient clinics and the Umeå University campus, Sweden. Inclusion criteria were: age 18–45 years, regular menstrual cycles (25–31 days), non-hormonal contraception. Participants were excluded if they were currently using steroid hormones and/or psychotropic or anti-depressant medication, if they had neurological conditions (e.g. multiple sclerosis, epilepsy, stroke), histories of traumatic brain injury, congenital and acquired intellectual disabilities; severe vision or hearing impairments; endocrine disorders (e.g. hypo/hyperthyroidism), gynecological disorders (e.g. polycystic ovarian syndrome), or psychiatric conditions (e.g. current mood, anxiety, psychotic and personality disorders), drug or alcohol abuse, and if they were pregnant or had contraindications for MRI. Prior to screening, a wash-out period of three months was required for psychotropic drugs, and at least one month for steroid hormones, along with normalization of menstrual cycling (e.g. following discontinuation of hormonal contraception). Potential participants were screened for psychiatric conditions (past and current) using the Swedish version of the Mini International Neuropsychiatric Interview (MINI) questionnaire [21], in order to exclude potential comorbid conditions or premenstrual exacerbation of other psychiatric disorders. However, due to their high prevalence, major depressive episodes and eating disorders in remission for more than two years prior to the study were allowed. Somatic conditions with potential effects on menstrual endocrinology or grey matter volume were screened for through detailed medical and gynecological histories and physical health assessments, conducted by the study’s lead physician (MB). All participants completed daily ratings of PMDD symptoms for a minimum of two menstrual cycles using the Daily Record of Severity of Problems (DRSP) [22], implemented in Swedish via an ad-hoc web platform. The DRSP is a validated scale for assessing PMDD, widely used in academic research and as a primary outcome measure in clinical trials [23, 24]. It demonstrates strong reliability, with a Cronbach’s α of 0.95, and a test-retest intraclass correlation of 0.83 for total symptoms [22]. All participants provided written informed consent to participate in the study. The study was approved by the Regional Ethical Review Board in Umeå (2016–111-31 M, 2017–266-32 M) and conducted in accordance with the Declaration of Helsinki.

A PMDD diagnosis, according to the Diagnostic and Statistical Manual of Mental Disorders, 5th Ed (DSM-5) [1], was confirmed using the algorithm developed by Endicott et al. [22], based on the following criteria: *i)* average daily symptom score ≤ 3 (“mild”) during the mid-follicular phase (days +6 to + 10 after the onset of menses); *ii)* during the late-luteal phase (days −5 to −1 prior to the onset of menses), at least two days with ratings ≥ 4 (“moderate”) for a minimum of one “core” mood symptom and at least five symptoms in total; *iii)* symptoms in the late-luteal phase interfered with daily functioning, which was defined as ratings of ≥ 4 for two days on at least one impairment item. A diagnosis of PMDD was given if the above criteria were met for two consecutive menstrual cycles and if the assessment of the daily ratings were in agreement with the clinical judgement of the investigator. Participants included in the control group were required to be asymptomatic across the entire menstrual cycle, i.e., no mean ratings > 3 during either phase.

### Procedures

**Fig. 1:**
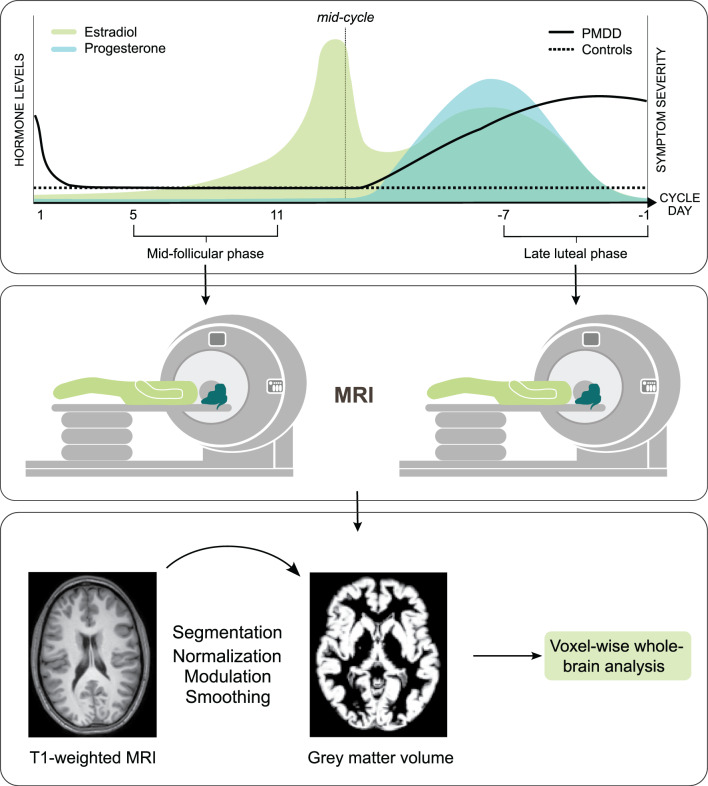
Study design. Individuals with PMDD and healthy controls were assessed twice; once in the asymptomatic mid-follicular phase (day 5 to day 11 after the previous menses) and once during the symptomatic late luteal phase (day −7 to − 1 before the next menses) of the menstrual cycle. The upper panel characterizes ovarian hormone fluctuations across the menstrual cycle and the timing and severity of PMDD symptoms (idealized data). Structural MRI brain data was collected on both occasions, to measure grey matter structure throughout the cycle. The brain images were preprocessed using the ‘segment’ routine of SPM12 and an 8-mm full-width half-maximum (FWHM) Gaussian kernel smoothing to generate parametric maps of grey matter volumes. Voxel-wise exploratory analyses of these maps were conducted in SPM12 to detect group-by-phase interaction effects on grey matter volumes throughout the brain. The main effects of group (PMDD versus controls) and phase (mid-follicular versus late luteal) were also assessed. Abbreviations: MRI, Magnetic Resonance Imaging; PMDD, PreMenstrual Dysphoric Disorder

Participants were scanned twice (Fig. 1); once in the mid-follicular phase (menstrual cycle day +5 to +11), and once in the late-luteal phase (menstrual cycle day − 7 to −1). Menstrual cycle phase was confirmed by self-reported previous and next menses, as well as serum concentrations of estradiol and progesterone. To verify that ovulation had occurred, serum concentration of progesterone during the luteal phase had to lie within two standard deviations of the progesterone standard-curve for the corresponding luteal phase day [25]. In addition, when participants were scanned in the last days of the cycle, the serum concentration of allopregnanolone, progesterone metabolite, was used to further confirm ovulation. Serum concentrations of progesterone and estradiol were determined using Elecsys^®^ Gen III immunoassays, detecting progesterone from 0.05 ng/ml and estradiol from 5 pg/ml. Serum concentrations of allopregnanolone were quantified using ultra-high-performance liquid chromatography mass spectrometry (UPLC-MS/MS), with a limit of quantification of 0.2 nM. All hormonal data were used to ensure compliance with the study design.

### MR acquisition

T1-weighted whole-brain (including cerebellum and brain stem) scans were collected using a 3.0 Tesla Discovery MR750 scanner (General Electric, Madison, WI, USA) equipped with a 32-channel head coil. The 3D fast spoiled gradient echo sequence included the following acquisition parameters: 8.2 ms repetition time, 3.2 ms echo time, 512 × 512 matrix size, 12° flip angle, 176 transversal slices, 0.48 × 0.48 × 1 mm^3^ voxel size, no fat suppression, 8:11 min duration.

### Voxel-based morphometry

Statistical Parametric Mapping (SPM12, version 7771, Welcome Trust Centre for Neuroimaging, University College London, UK) implemented in MATLAB R2019b (version 9.7.0.1586710, MathWorks, Natick, MA, USA), and running on Windows 10 was used for pre-processing and analysis of structural MRI data. The voxel-based morphometry (VBM) preprocessing steps were carried out as previously described [11, 26]. The final quality check resulted in the exclusion of one participant detected as an outlier ( > 2x standard deviations), whose follicular grey matter map was cropped dorsally.

The present VBM data only partly overlaps with those of a previous publication based on a larger dataset built by merging data from two neuroimaging studies done in Sweden. The aims, study population, and assessment time points of the present work differ from a previous publication on brain volumes, which only included luteal phase scans, and was focusing on the difference between individuals with PMDD and healthy controls during the symptomatic phase of the menstrual cycle [11]. Participants included in the present analysis represent 41% of women with PMDD and 62% of healthy controls included in this previous publication. The research question here investigated (whether volumetric alterations in PMDD vary across the menstrual cycle) substantively differs from the hypothesis tested in the previous publication. More recently, two reports based on the present sample of participants were published, however including measures of cortical architecture (surface-based morphometry) [27] and brain function [28], which do not overlap with the voxel-based morphometry data presented here.

### Statistical analyses

Group-by-Phase interaction effects on GMV were assessed by a whole-brain voxel-wise exploratory analysis carried out in SPM12, based on the general linear model using a flexible factorial design (repeated-measures analysis of variance). The design matrix was built using Group (PMDD, controls) as between-subject factor, with Phase (mid-follicular, late-luteal) and Subjects as within-subject factors. Post-hoc unpaired and paired-t-tests (two-tailed) were run to assess the main effect of Group (across phases) and Phase (across groups), respectively. Age, BMI and TIV were included as co-variates in the following post-hoc analyses comparing groups in each phase, considering their significant impact on volumetric measures [29, 30]. These voxel-wise analyses were run within a mask of grey matter defined by an absolute threshold set at 0.2, and statistical significance was set at *p* < 0.1, Family-Wise Error (FWE)-corrected using Threshold-Free Cluster Enhancement (TFCE) [31]. The use of a more permissive statistical significance threshold aims to reduce the risk of Type II errors, particularly in cases where effect sizes are not small. This approach is appropriate given the novel and exploratory nature of the study, allowing for the detection of potentially meaningful effects that warrant further investigation. Effect size maps were generated using the CAT12 toolbox. Both average effect sizes within clusters and effect sizes at peak voxels are reported, in the text and the supplementary tables, respectively.

Statistical significance was set as *p* < 0.05, with trend-level findings being described at the *p* < 0.1 threshold.

## Results

### Participants’ characteristics

**Fig. 2:**
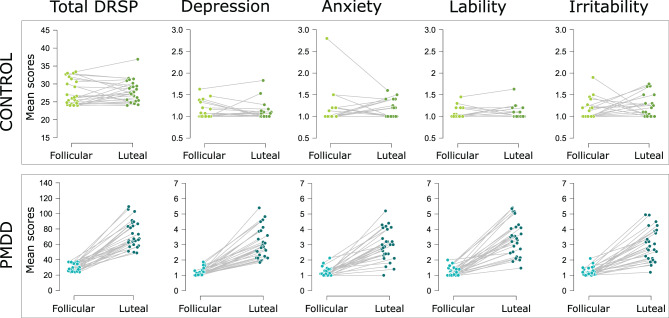
Change in DRSP scores from the mid-follicular to the late luteal days during screening. The distribution of individual symptom ratings across menstrual cycle phases during the screening period, and within each group is illustrated. The average mid-follicular (day +6 to day +10) and late luteal (day −5 to day −1) DRSP scores are given for the sum of all DRSP symptom items (total DRSP), and for each of the four core PMDD symptoms (depression, anxiety, affective lability, irritability). The depression score illustrates the mean ratings across the DRSP items “felt depressed”, “felt hopeless”, and “felt guilty”. The anxiety score illustrates the mean ratings of the DRSP item “felt anxious or tensed. The affective lability score illustrates the mean ratings across the DRSP items “had mood swings”, and “was more sensible to rejection or easily hurt”. The irritability score illustrates the mean ratings across the DRSP items “felt angry, irritable”, and “had conflicts with people”. The PMDD group displayed greater symptom severity compared to the control group, especially in the late luteal phase. Compared to the mid-follicular phase, DRSP scores were significantly increased in the late luteal phase of individuals with PMDD (*p*_Bonferroni_ < 0.001), while healthy controls did not display any significant change in symptom severity

The demographic, clinical and endocrine characteristics of the participants included in the study are presented in Table 1. Due to withdrawal of consent (*n* = 3), anovulation/early luteal scan (*n* = 4), booking errors (*n* = 1), screening failure (*n* = 1) and a cropped image (*n* = 1), a total of ten participants (4 PMDD, 6 controls) were excluded from the analyses, which included 28 individuals with PMDD and 26 controls. The groups did not differ in terms of age, BMI, TIV, menstrual cycle length, serum levels of ovarian hormones and menstrual cycle day at scanning (Table 1). They did not differ in terms of parity and psychiatric history, although individuals with PMDD tended to report past depression or eating disorder more often than controls (Table 1). While the DRSP symptom ratings in the control group did not significantly change between the mid-follicular and the late luteal phases, the PMDD group displayed a substantial increase in symptom severity (Fig. 2).

**Table 1:** Participants’ characteristics

	Controls (*N* = 26)		PMDD (*N* = 28)		Group difference
	Mean (SD) or N (%)	Range	Mean (SD) or N (%)	Range	p-value
Demographics					
Age (years)	28.0 (5.7)	21–40	28.6 (6.2)	18–41	0.52^a^
BMI (kg/m^2^)	24.3 (4.2)	18.6–33.8	23.6 (3.3)	18.4–29.6	0.54
TIV (L)	1.5 (0.1)	1.3–1–7	1.5 (0.1)	1.3–1–7	0.51
Menstrual cycle length (days)	28.7 (1.9)	26–36	27.7 (1.9)	21–31	0.13^a^
Parity (nulliparous)	21 (81.0)		19 (68.0)		0.46^b^
Psychiatric history^d^	3 (11.5)		9 (32.1)		0.10^c^
Depression	3 (11.5)		8 (28.6)		0.18^c^
Eating disorder	0		1 (3.6)		1.00^c^
Premenstrual symptoms^e^					
*Mid-follicular phase*					
Total DRSP score	27.3 (3.5)	24.0–33.4	29.9 (4.9)	24.0–37.2	0.03 ^a^
Depression DRSP items score	1.1 (0.2)	1.0–1.6	1.2 (0.3)	1.0–1.9	0.02 ^a^
Anxiety DRSP item score	1.1 (0.4)	1.0–2.8	1.2 (0.3)	1.0–2.1	0.04 ^a^
Lability DRSP items score	1.1 (0.1)	1.0–1.4	1.2 (0.3)	1.0–2.0	3.0 e-3 ^a^
Irritability DRSP items score	1.1 (0.2)	1.0–1.9	1.2 (0.3)	1.0–2.1	0.01 ^a^
*Late luteal phase*					
Total DRSP score	27.9 (3.0)	24.0–36.9	72.7 (16.9)	48.8–109.1	2.9 e-10 ^a^
Depression DRSP items score	1.1 (0.2)	1.0–1.8	3.0 (1.0)	1.8–5.4	1.9 e-10 ^a^
Anxiety DRSP item score	1.1 (0.2)	1.0–1.6	3.0 (1.0)	1.0–5.2	1.9 e-9 ^a^
Lability DRSP items score	1.1 (0.1)	1.0–1.6	3.4 (1.1)	1.5–5.4	1.5 e-10 ^a^
Irritability DRSP items score	1.2 (0.2)	1.0–1.7	3.0 (1.0)	1.2–4.9	1.3 e-9 ^a^
Hormones					
*Mid-follicular phase*					
Test day	+7.7 (1.4)	+5 – +10	+8.0 (1.9)	+5 – +11	0.57^a^
Progesterone (nmol/L)	0.8 (0.9)	0.3–4.5	0.7 (0.5)	0.2–2.0	0.80^a^
Estradiol (pmol/L)	278 (218)	69–996	301 (273)	27–1295	0.89^a^
*Late luteal phase*					
Test day	−3.8 (1.7)	−7 – −1	−4.3 (1.9)	−7 – −1	0.42^a^
Progesterone (nmol/L)	23.4 (13.6)	1.4–53.0	23.5 (14.0)	1.2–57.0	0.96^a^
Estradiol (pmol/L)	414 (188)	163–988	445.8 (226)	27–1001	0.96^a^

BMI = body mass index, TIV = total intracranial volume, PMDD = premenstrual dysphoric disorder. * ** The average mid-follicular (day +6 to day +10) and late luteal (day -5 to day -1) DRSP scores are given for the sum of all DRSP symptom items (total DRSP), and for each of the four core PMDD symptoms (depression, anxiety, affective lability, irritability). The depression score illustrates the mean ratings across the DRSP items “felt depressed”, “felt hopeless”, and “felt guilty”. The anxiety score illustrates the mean ratings of the DRSP item “felt anxious or tensed. The affective lability score illustrates the mean ratings across the DRSP items “had mood swings”, and “was more sensible to rejection or easily hurt”. The irritability score illustrates the mean ratings across the DRSP items “felt angry, irritable”, and “had conflicts with people”. Group comparisons were conducted using Student t-tests unless otherwise specified. a non-parametric testing using Mann-Whitney U Test. b Pearson Chi-Square test. c Fisher Exact test, d Psychiatric history based on the Mini International Neuropsychiatric Interview (MINI) questionnaire. e Severity of premenstrual symptoms assessed during the screening cycles, before the experiment

### State vs. trait grey matter characteristics of PMDD

**Fig. 3:**
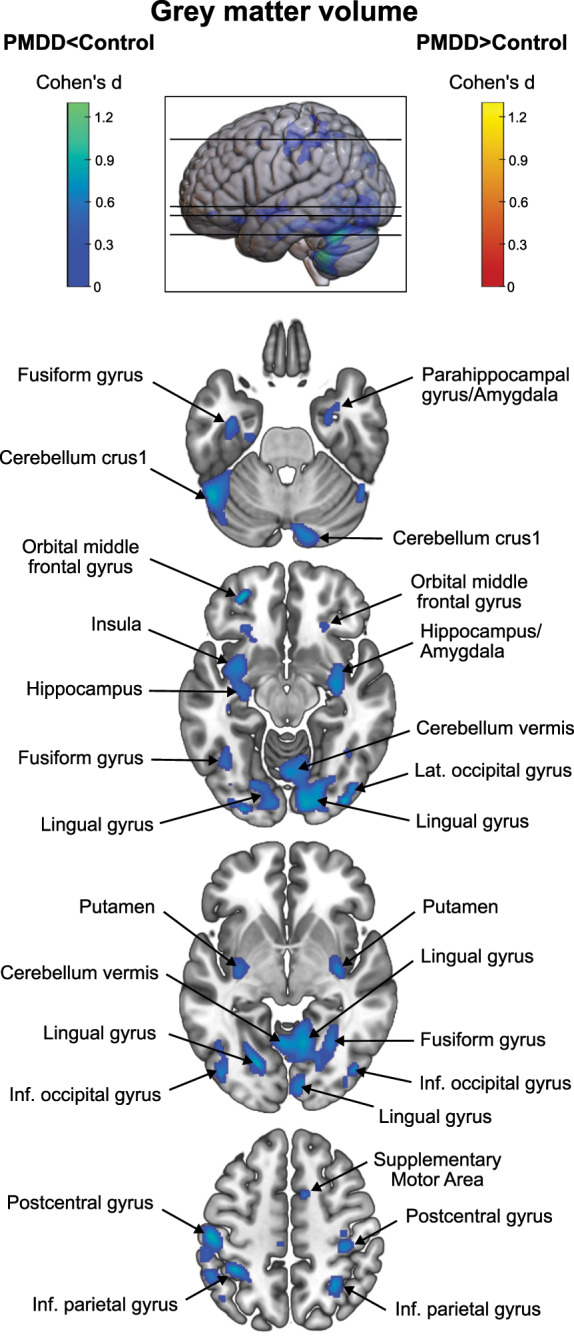
Main effect of group on grey matter volume. The main effect of group (PMDD versus controls) on grey matter volume was assessed over the whole brain, across menstrual cycle phases. The results indicate smaller volumes in cerebellar, occipital, ventral occipito-temporal, parietal, paracentral and orbitofrontal areas, and in the putamen, in individuals with PMDD compared to healthy controls (*p* < 0.1, family-wise error (FWE) corrected). The significant and trend-level clusters are shown in blue over axial brain slices taken at the following z coordinates: −28, −12, −5, 50. The color bars indicate Cohen’s d effect sizes within the clusters using a threshold of *p* < 0.1 following family-wise error (FWE)-correction. Grey matter regions were labelled based on the automated anatomical labelling (AAL) atlas. No significant difference was found in the opposite direction of effect. Cohen’s *d* maps were generated in SPM12 and brain figures were generated using MRIcroGL (v1.2.20200331). Abbreviations: Inf., inferior; Lat., lateral

At the whole-brain level, no interaction between group and menstrual cycle phase was observed on GMV (*p*_FWE_ > 0.1, TFCE). Pointing to trait- rather than state structural markers of PMDD, a main effect of group was found across menstrual cycle phases. Smaller volumes were present in individuals with PMDD compared to healthy controls, primarily in the cerebellum and cuneus (*p*_FWE_ < 0.05, TFCE, Fig. 3, Table S1), and less significantly in ventral occipital, temporal (including hippocampus and amygdala), parietal, paracentral and orbitofrontal areas, and putamen (p_FWE_ < 0.1, TFCE, Fig. 3, Table S1). Group comparisons conducted in each phase yielded very similar findings, showing smaller volumes in PMDD compared to controls (Tables S2 and S3). Effect sizes suggest medium effects across the observed clusters (average Cohen’s *d* = 0.6). No effect of menstrual cycle phase on GMV was observed.

## Discussion

The current study suggests that individuals with PMDD display stable, trait-like differences in grey matter structure compared with controls, independently of short-term fluctuations in endogenous ovarian hormones. More specifically, individuals with PMDD had smaller GMV in cerebellar and occipital areas, with indications of potential effects in occipital, temporal, parietal, paracentral, and orbitofrontal areas, as well as in the putamen, across the menstrual cycle. The results align with and expand upon findings from the largest structural MRI study of PMDD to date, which reported smaller GMV in similar regions during the symptomatic luteal phase [11]. Our findings suggest that these GMV differences may not be exclusive to the premenstrual phase of the menstrual cycle.

The present findings partly overlap with brain regions highlighted by functional neuroimaging studies of emotion processing in PMDD, particularly the amygdala, ventral striatum, prefrontal, parietal and paracentral regions [14]. Among these, the amygdala, ventral striatum, and orbitofrontal cortex constitute key hubs of emotional networks [32]. On the other hand, clusters of smaller GMV were also found in temporal and occipital cortices (particularly the fusiform and lingual gyri) as well as in the cerebellum, regions not typically associated with emotional processing in functional neuroimaging studies of PMDD. The use of a sole *a priori* region-of-interest approach and the small sample sizes of available fMRI studies in PMDD may partly explain the lack of findings in these regions [14]. Nevertheless, the lingual and fusiform gyri are involved in face processing [33], and activity in these regions is modulated by the amygdala during the perception of facial expressions of emotions [34]. Furthermore, variations in grey matter structure in these regions have previously been associated with the severity of depressive and anxious symptoms in other psychiatric disorders [35–37], as well as altered processing of information in the visual cortex, which interferes with the recruitment of attentional neuronal networks, in major depressive disorder [38]. The cerebellum, on the other hand, is engaged in a wide variety of motor, cognitive and emotional functions, and is hypothesized to be involved in the perception, recognition, and evaluation of emotion, as well as its integration into behavior [39]. Interestingly, structural and functional alterations have been found in the cerebellum of patients with other depressive disorders, such as bipolar and unipolar depression [39]. In PMDD, a greater activity of the emotional cerebellum was observed in patients relative to controls during a cognitive memory task [40], and one study reported associations between increased cerebellar metabolic activity from the follicular to the luteal phase and deterioration of mood [41]. Furthermore, our finding in the cerebellum is in line with a previous study specifically examining the luteal-phase GMV differences between PMDD patients and healthy controls (*N* = 131) [11]. Conversely, one smaller study (N_PMDD_ = 12, N_control_ = 13) previously reported larger cerebellar GMV in PMDD (driven by an age-dependent reduction in cerebellar GMV in controls), located in slightly different sub-regions of the cerebellum [7]. Participants in the present sample were young, and age-related reductions in cerebellar GMV could not be detected; this, in combination with differences in analytical approach and sample size, may explain the disparity between the present result and this earlier study. Nonetheless, structural alterations in limbic, visual and cerebellar networks may contribute to, or result from, abnormal emotion processing, and may help explain the affective symptoms experienced by individuals with PMDD. In sum, the observation of smaller GMV in PMDD, as well as in other mood disorders with overlapping symptoms, raises important questions about the ontogeny and pathophysiology of these conditions. While such structural differences may reflect a shared vulnerability to affective dysregulation, their role in a hormone-specific disorder like PMDD remains unclear.

Notably, the biological underpinnings of GMV variations are largely unknown. Differences in GMV are hypothesized to be mainly related to glial cell morphology, synaptic and dendritic spine density and vascular volume [42]. Further research examining whether differential brain structure in PMDD is stable across the lifespan (i.e., vulnerability factor), whether it is influenced by PMDD symptom severity (i.e., impact of the disorder on the brain), and if they are reversed by effective treatment (i.e., reversible), could provide valuable insights about the driving factors underlying brain structural correlates of vulnerability in individuals with PMDD.

In contrast to our trait-like structural findings, previous studies have shown that variations in brain function, metabolism and neurotransmission in PMDD occur across the menstrual cycle [14]. Significant group-by-menstrual cycle phase interaction effects, indicating that differences between PMDD patients and controls are specific to a particular menstrual cycle phase, have been reported in several brain regions (including the amygdala, insula, prefrontal, occipital and cerebellar regions) based on task-based fMRI studies and molecular imaging studies investigating brain-glucose metabolism, serotonergic transmission, and $\gamma$-aminobutyric acid (GABA) transmission [14]. Conversely, other studies have identified phase-independent, trait-like differences between individuals with PMDD and controls, notably on the neural recruitment of the anterior cingulate and ventromedial PFC during an emotional task [43], and the brain functional connectivity [44, 45]. Thus, while we found that structural alterations of grey matter in PMDD seem to be relatively stable across the menstrual cycle, functional studies point to both hormone-dependent and hormone-independent effects, highlighting the complex relationship between brain structure and function. Interestingly, a resting-state fMRI study showed that temporo-occipital and fronto-striatal functional connections across the cycle mediate the relationship between difficulties in regulating emotions and PMDD diagnosis [44], suggesting long-lasting alterations in functional networks involving brain areas where we found trait-like differences in GMV in individuals with PMDD. The present findings are also in line with recent studies highlighting the efficacy of selective progesterone receptor modulation in individuals with PMDD [46], without any impact on grey matter volume [47]. The absence of structural changes, despite this treatment efficacy in alleviating symptoms, suggests that these differences may not be directly tied to the acute pathophysiology of PMDD. Instead, they could represent a predisposing trait or a consequence of recurrent cyclical distress. Future research should explore whether these structural differences influence sensitivity to hormonal fluctuations or interact with functional brain changes to mediate PMDD symptomatology. Furthermore, the field would benefit from examining when these trait changes emerge in relation to shifts in ovarian hormone levels (e.g., menarche, pregnancy) by recruiting larger, more age-diverse samples of women.

The present investigation displays several strengths in the context of the current neuroimaging literature on PMDD, such as the use of the prospective DRSP questionnaire for selection of both PMDD and control groups, and the use of both cycle mapping and hormonal assessment to confirm the menstrual cycle phase. Given the uniqueness of this dataset, we opted to present results at the relatively lenient threshold (*p*_FWE_ < 0.10, TFCE) in order to illustrate trends that may guide future works. At the same time, some limitations should be acknowledged. The modest sample size reduces statistical power, particularly for detecting small effects, and our study is therefore best positioned to detect medium-to-large effects (here observed in the left cerebellum and cuneus). This limitation aligns with recent concerns regarding reproducibility in small-sample neuroimaging research [48]. Nevertheless, menstrual-cycle–related shifts in brain volumes have been reported using comparable sample sizes [17], and even in dense sampling studies of a single participant [49, 50], confirming that our cohort was sufficiently powered to detect potential cycle-dependent effects. Additionally, our within-subject design reduces between-subject variability and improves sensitivity, and our focus on a clinically defined, prospectively verified PMDD sample provides mechanistic insights that complement large-scale population-based studies. We therefore view our findings as preliminary but valuable, warranting replication in larger, ideally multi-site or consortia-based studies. Of note, associations between ovarian hormone levels and GMV were not examined, as hormone measurements were limited to a single time point in the menstrual cycle, which is insufficiently informative. Furthermore, the low contrast between tissues within subcortical structures in MR images may impact the quality of segmentation by automated pipelines [51], and findings in the amygdala should thus be viewed with particular caution. Although non-significant in the present sample, the difference in past history of psychiatric symptoms (primarily depressive) between individuals with PMDD and healthy controls, could represent a potential confound in our analyses. However, adjusting our analyses for this variable did not substantially change the direction and anatomical location of the presented findings (Tables S6 and S7).

## Conclusions

By comparing the brain structure of individuals with PMDD during the mid-follicular and the late luteal phases of the menstrual cycle, we sought to assess the neural correlates of the on and off switch of mood symptoms defining PMDD. The findings provide the first evidence for an altered grey matter structure in the brains of individuals with PMDD across the symptomatic and asymptomatic phases of the menstrual cycle, which might represent trait-like characteristics of the PMDD brain. Further investigations are needed to elucidate whether the observed smaller volumes in individuals with PMDD constitute stable vulnerability markers or whether they gradually develop over the course of the disorder. These new findings suggest that both trait-like differences in brain structure and state-like differences in brain reactivity to fluctuating ovarian hormone levels may contribute to the pathophysiology of PMDD. This dual mechanism framework highlights the need for further research to disentangle the relative contributions of stable neurobiological traits and dynamic hormone-sensitive changes in brain function, which could inform more targeted therapeutic strategies.

## Electronic supplementary material

Below is the link to the electronic supplementary material.

Supplementary Material 1

## Data Availability

The datasets used and/or analysed during the current study are available from the corresponding author on reasonable request.

## Abbreviations

AAL: Automated Anatomical Labeling (atlas)
ANOVA: Analysis of Variance
BMI: Body Mass Index
CAT: Computational Anatomy Toolbox
DRSP: Daily Record of Severity of Problems
DSM-5: Diagnostic and Statistical Manual of Mental Disorders, Fifth Edition
FDR: False Discovery Rate
fMRI: Functional Magnetic Resonance Imaging
FWE: Family-Wise Error
GMV: Grey Matter Volume
MRI: Magnetic Resonance Imaging
MINI: Mini International Neuropsychiatric Interview
PMDD: Premenstrual Dysphoric Disorder
SPSS: Statistical Package for the Social Sciences
SPM12: Statistical Parametric Mapping version 12
TFCE: Threshold-Free Cluster Enhancement
TIV: Total Intracranial Volume
UPLC-MS/MS: Ultra-High-Performance Liquid Chromatography–Tandem Mass Spectrometry
VBM: Voxel-Based Morphometry
