# Supplementary material for: Trait- versus state- grey matter volume alterations in premenstrual dysphoric disorder
Source: BMC Psychiatry. 2025 Dec 2;25:1139. doi: 10.1186/s12888-025-07533-5 (PMC12670873; doi:10.1186/s12888-025-07533-5)
Supplement: Supplementary file 1 — Supplementary Material 1 [file 12888_2025_7533_MOESM1_ESM.docx]

# Supplemental Information

#
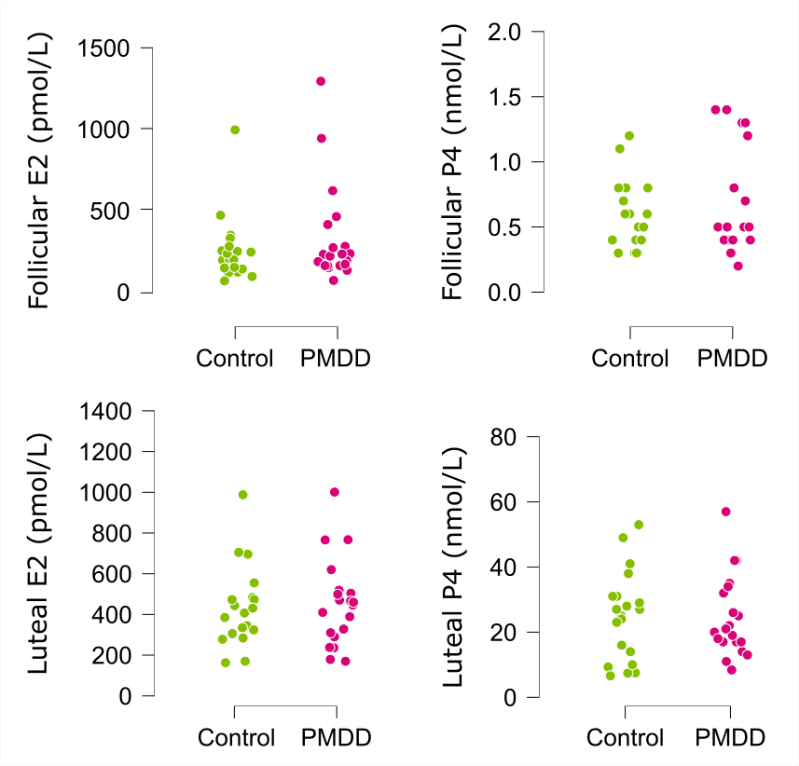
Figure S1. Ovarian hormones levels. The distribution of individual serum estradiol (E2) and progesterone (P4) concentrations across menstrual cycle phases and within each group is illustrated. No significant group difference was found in the mean hormone levels, nor in the inter-individual variance of hormone levels (Levene’s test *p*>0.05).


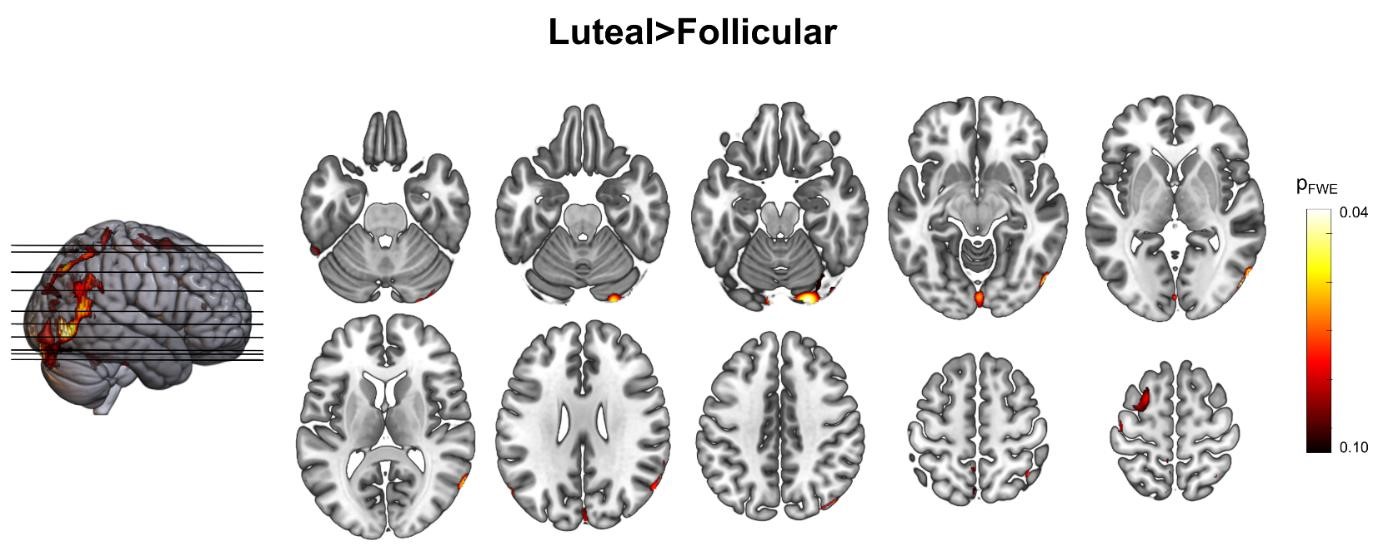
**Figure S2. Main effect of phase across groups.** The Main effect of Phase (mid-follicular versus late luteal) on grey matter volume was assessed over the whole brain, across women with PMDD and healthy controls. The results indicate smaller volumes in the mid-follicular phase compared to the late luteal phase (p<0.1, Family-Wise Error (FWE) corrected), although clearly showing edge effects. The significant clusters are shown in red-yellow over axial brain slices taken at the following z coordinates: -27, -23, -20, -10, 0, 10, 26, 40, 56, 61. The color bars indicate statistical significance with a threshold of p<0.1 following Family-Wise Error (FWE)-correction. Post-hoc comparisons did not show any significant difference between the mid-follicular and the late luteal phase in each group separately. No significant difference was found in the opposite direction of effect. Brain figures were generated using MRIcroGL (v1.2.20200331).

**Table S1. Main effect of group on grey matter volume over the whole brain, across menstrual cycle phases.**

|  | **Cluster size** | **p_FWE_** | **Cohen’s d** | **MNI coordinates** | | | **AAL region** |
| --- | --- | --- | --- | --- | --- | --- | --- |
|  |  |  |  | **x** | **y** | **z** |  |
| ***CTRL > PMDD*** | | | | | | | |
|  | 5312 | 0.041 | 0.96 | -41 | -45 | -45 | Cerebellum 7b L |
|  |  | 0.047 | 0.90 | -48 | -56 | -30 | Cerebellum Crus1 L |
|  |  | 0.056 | 0.55 | -30 | -47 | -59 | Cerebellum 8 L |
|  |  | 0.061 | 0.68 | -39 | -59 | -20 | Fusiform L |
|  |  | 0.064 | 0.66 | -20 | -72 | -2 | Lingual L |
|  |  | 0.069 | 0.68 | -42 | -62 | -8 | Temporal Inf L |
|  |  | 0.080 | 0.53 | -23 | -36 | -44 | Cerebellum 10 L |
|  |  | 0.080 | 0.39 | -29 | -92 | -12 | Occipital Inf L |
|  | 289 | 0.048 | 1.14 | -12 | -87 | 36 | Cuneus L |
|  |  | 0.083 | 0.46 | -9 | -80 | 42 | Occipital Sup L |
|  | 3836 | 0.051 | 0.86 | 12 | -89 | -9 | Lingual R |
|  |  | 0.054 | 0.75 | 3 | -68 | -11 | Vermis 6 |
|  |  | 0.056 | 0.76 | -2 | -60 | -5 | Vermis 4 5 |
|  |  | 0.056 | 0.37 | 12 | -86 | 8 | Calcarine R |
|  |  | 0.064 | 0.73 | 12 | -84 | -26 | Cerebellum Crus1 R |
|  |  | 0.074 | 0.56 | 17 | -83 | 35 | Occipital Sup R |
|  |  | 0.078 | 0.44 | 29 | -53 | -2 | Fusiform R |
|  |  | 0.080 | 0.50 | 11 | -87 | 30 | Cuneus R |
|  | 972 | 0.063 | 0.96 | -50 | -21 | 48 | Postcentral L |
|  |  | 0.081 | 0.83 | -38 | -60 | 54 | Parietal Inf L |
|  |  | 0.090 | 0.66 | -56 | -26 | 33 | SupraMarginal L |
|  | 484 | 0.065 | 0.96 | -35 | -42 | 47 | Parietal Inf L |
|  |  | 0.081 | 0.68 | -27 | -47 | 54 | Postcentral L |
|  |  | 0.090 | 0.61 | -20 | -47 | 62 | Parietal Sup L |
|  | 652 | 0.067 | 0.87 | 42 | -47 | -45 | Cerebellum 7b R |
|  |  | 0.081 | 0.69 | 27 | -41 | -45 | Cerebellum 10 R |
|  |  | 0.098 | 0.52 | 36 | -51 | -57 | Cerebellum 8 R |
|  | 177 | 0.074 | 0.89 | -21 | 23 | -17 | Frontal Inf Orb L |
|  | 264 | 0.074 | 0.88 | 0 | -21 | 59 | Supp Motor Area L |
|  |  | 0.086 | 0.66 | -5 | -27 | 54 | Paracentral Lobule L |
|  | 240 | 0.075 | 0.85 | 29 | -53 | 53 | Parietal Inf R |
|  | 458 | 0.076 | 0.79 | 33 | -14 | -9 | Putamen R |
|  | 208 | 0.078 | 0.83 | 38 | -87 | -11 | Occipital Inf R |
|  | 692 | 0.085 | 0.65 | -32 | 0 | -11 | N.A.^a^ |
|  | 53 | 0.088 | 0.84 | -29 | 47 | -12 | Frontal Mid Orb L |
|  | 99 | 0.089 | 0.74 | 23 | 24 | -17 | Frontal Inf Orb R |
|  | 210 | 0.090 | 0.68 | 39 | -59 | -18 | Fusiform R |
|  | 100 | 0.092 | 0.69 | 26 | -8 | -32 | ParaHippocampal R |
|  | 146 | 0.093 | 0.69 | 36 | -27 | 50 | Postcentral R |
|  |  | 0.093 | 0.69 | 35 | -11 | 47 | Precentral R |
|  | 34 | 0.094 | 0.75 | 11 | 6 | 48 | Supp Motor Area R |
|  | 61 | 0.097 | 0.63 | 48 | -54 | -30 | Cerebellum Crus1 R |
|  | 25 | 0.097 | 0.67 | 21 | 2 | 60 | Frontal Sup R |
| ***CTRL < PMDD*** | | | N.S. | | | | |

All peak p-values are derived from Threshold-Free Cluster Enhancement (TFCE). Redundant peaks inside clusters, and clusters including less than 5 voxels are not listed. ^a^ The peak coordinates of this cluster did not fall into any grey matter label according to the AAL atlas, however, the cluster covered parts of the left putamen, insula, hippocampus, superior temporal gyrus and parahippocampal gyrus. AAL, Automatic Anatomical Labelling atlas; FWE, Family Wise Error correction; PMDD, premenstrual dysphoric disorder; CTRL, healthy controls; L, left; R, right; N.S., non-significant.

**Table S2. Group comparison of grey matter volume over the whole brain, in the follicular phase of the menstrual cycle.**

|  | **Cluster size** | | **p_FWE_** | **MNI coordinates** | | | **AAL region** |
| --- | --- | --- | --- | --- | --- | --- | --- |
|  |  |  |  | **x** | **y** | **z** |  |
| ***CTRL > PMDD*** | | | | | | | |
|  | 2631 | | 0.053 | -41 | -50 | -47 | Cerebellum 7b L |
|  |  | | 0.056 | -42 | -57 | -44 | Cerebellum Crus2 L |
|  |  | | 0.063 | -29 | -48 | -56 | Cerebellum 8 L |
|  |  | | 0.068 | -48 | -56 | -32 | Cerebellum Crus1 L |
|  |  | | 0.085 | -38 | -57 | -20 | Fusiform L |
|  |  | | 0.094 | -39 | -50 | -27 | Cerebellum 6 L |
|  | 156 | | 0.062 | -51 | -20 | 48 | Postcentral L |
|  | 1066 | | 0.063 | 27 | -56 | -3 | ParaHippocampal R |
|  |  | | 0.084 | 33 | -9 | -8 | Putamen R |
|  | 703 | | 0.075 | 12 | -56 | -3 | Lingual R |
|  |  | | 0.091 | 3 | -68 | -11 | Vermis 6 |
|  |  | | 0.094 | -2 | -60 | -5 | Vermis 4 5 |
|  | 509 | | 0.075 | 42 | -53 | -47 | Cerebellum 7b R |
|  | 313 | | 0.088 | 12 | -90 | -9 | Lingual R |
|  | 137 | | 0.090 | 17 | -84 | -27 | Cerebellum Crus1 R |
|  | 125 | | 0.091 | -8 | -59 | -44 | Cerebellum 9 L |
|  | 69 | | 0.093 | 12 | -86 | 8 | Calcarine R |
|  | 107 | | 0.094 | -23 | -24 | -23 | ParaHippocampal L |
|  | 31 | | 0.094 | -8 | -84 | 35 | Cuneus L |
|  | 204 | | 0.095 | -36 | -6 | -6 | Insula L |
|  |  | | 0.097 | -29 | 2 | -17 | Amygdala L |
|  | 93 | | 0.095 | 39 | -60 | -18 | Fusiform R |
|  | 53 | | 0.096 | -26 | 23 | -18 | Frontal Inf Orb L |
|  | 35 | | 0.098 | 24 | -39 | -47 | Cerebellum 10 R |
|  | 14 | | 0.098 | 11 | -87 | 29 | Cuneus R |
| ***CTRL < PMDD*** | | N.S. | | | | | |

All peak p-values are derived from Threshold-Free Cluster Enhancement (TFCE). Redundant peaks inside clusters are not listed. AAL, Automatic Anatomical Labelling atlas; FWE, Family Wise Error correction; PMDD, premenstrual dysphoric disorder; CTRL, healthy controls; L, left; R, right; N.S., non-significant.

**Table S3. Group comparison of grey matter volume over the whole brain, in the luteal phase of the menstrual cycle.**

|  | **Cluster size** | | **p_FWE_** | **MNI coordinates** | | | **AAL region** |
| --- | --- | --- | --- | --- | --- | --- | --- |
|  |  |  |  | **x** | **y** | **z** |  |
| ***CTRL > PMDD*** | | | | | | | |
|  | 3384 | | 0.045 | -39 | -47 | -47 | Cerebellum 8 L |
|  |  | | 0.048 | -42 | -54 | -42 | Cerebellum Crus2 L |
|  |  | | 0.050 | -48 | -56 | -32 | Cerebellum Crus1 L |
|  |  | | 0.058 | -39 | -59 | -20 | Fusiform L |
|  |  | | 0.061 | -41 | -50 | -26 | Cerebellum 6 L |
|  | 5576 | | 0.057 | 12 | -59 | -5 | Lingual R |
|  |  | | 0.069 | 12 | -86 | 9 | Calcarine R |
|  |  | | 0.071 | 17 | -83 | -29 | Cerebellum Crus1 R |
|  |  | | 0.071 | 42 | -51 | -47 | Cerebellum 7b R |
|  |  | | 0.072 | -2 | -60 | -5 | Vermis 4 5 |
|  |  | | 0.072 | 3 | -66 | -11 | Vermis 6 |
|  |  | | 0.075 | 8 | -83 | -14 | Cerebellum 6 R |
|  |  | | 0.078 | 17 | -86 | 36 | Occipital Sup R |
|  | 1700 | | 0.057 | 26 | -8 | -30 | ParaHippocampal R |
|  |  | | 0.064 | 32 | -9 | -9 | Putamen R |
|  |  | | 0.088 | 23 | 12 | -35 | Temporal Pole Sup R |
|  |  | | 0.093 | 32 | 14 | -17 | Insula R |
|  | 164 | | 0.073 | -8 | -83 | 35 | Cuneus L |
|  | 457 | | 0.086 | -35 | -6 | -6 | Insula L |
|  |  | | 0.087 | -32 | 0 | -9 | Putamen L |
|  | 528 | | 0.090 | 12 | -36 | -27 | Cerebellum 3 R |
|  |  | | 0.093 | 41 | -59 | -18 | Fusiform R |
|  |  | | 0.097 | 23 | -47 | -26 | Cerebellum 4 5 R |
|  | 9 | | 0.098 | -20 | -72 | -2 | Lingual L |
| ***CTRL < PMDD*** | | N.S. | | | | | |

All peak p-values are derived from Threshold-Free Cluster Enhancement (TFCE). Redundant peaks inside clusters are not listed. AAL, Automatic Anatomical Labelling atlas; FWE, Family Wise Error correction; PMDD, premenstrual dysphoric disorder; CTRL, healthy controls; L, left; R, right; N.S., non-significant.

**Table S4. Group-by-phase interaction and main effects on amygdala GMV**.

| **ROI** | **Group x Phase interaction** | | | **Main Phase effect** | | | **Main Group effect** | | |
| --- | --- | --- | --- | --- | --- | --- | --- | --- | --- |
|  | F | p | η_p_^2^ | F | p | η_p_^2^ | F | p | η_p_^2^ |
| Amygdala L | 0.874 | 0.354 | 0.017 | 0.003 | 0.953 | 6.6E-5 | 0.333 | 0.567 | 0.006 |
| Amygdala R | 0.016 | 0.899 | 3.1E-4 | 1.794 | 0.186 | 0.033 | 1.008 | 0.320 | 0.019 |

Repeated-measures ANOVA yielded negative results pointing to an absence of Group-by-Phase interaction effects on amygdala grey matter volumes (GMV). L = left, R = right. η_p_^2^ = partial eta square effect size.

**Table S5. Group effects on amygdala grey matter volume in each menstrual cycle phase.**

| **ROI** | **GROUP EFFECT** | | | | | | | |
| --- | --- | --- | --- | --- | --- | --- | --- | --- |
|  | **MID-FOLLICULAR** | | | | **LATE LUTEAL** | | | |
|  | Direction | F | p | η_p_^2^ | Direction | F | p | η_p_^2^ |
| Amygdala L | CTRL>PMDD | 2.53 | 0.118 | 0.049 | CTRL>PMDD | 1.58 | 0.214 | 0.031 |
| Amygdala R | CTRL>PMDD | 4.83 | 0.033* | 0.090 | CTRL>PMDD | 4.42 | 0.041* | 0.083 |

Post-hoc group comparison conducted in each phase revealed smaller grey matter volumes (GMV) in the right amygdala of women with PMDD compared to healthy controls. CTRL = healthy controls, L = left, PMDD = premenstrual dysphoric disorder, R = right. η_p_^2^ = partial eta square effect size. * p<0.05, uncorrected for multiple testing, p<0.1 after FDR correction.

**Table S6.** **Group comparison of grey matter volume over the whole brain, in the follicular phase of the menstrual cycle, after adjusting for psychiatric history.**

|  | **Cluster size** | | **p_FWE_** | **MNI coordinates** | | | **AAL region** |
| --- | --- | --- | --- | --- | --- | --- | --- |
|  |  |  |  | **x** | **y** | **z** |  |
| ***CTRL > PMDD*** | | | | | | | |
|  | 813 | | 0.077 | -40 | -50 | -46 | Cerebellum 8 L |
|  |  | | 0.082 | -42 | -57 | -44 | Cerebellum Crus2 L |
|  | 56 | | 0.080 | -51 | -20 | 48 | Postcentral L |
|  | 107 | | 0.084 | 27 | -9 | -30 | ParaHippocampal R |
|  | 135 | | 0.091 | 12 | -56 | -3 | Lingual R |
|  | 38 | | 0.097 | -50 | -54 | -32 | Cerebellum Crus1 L |
|  | 29 | | 0.098 | 42 | -52 | -46 | Cerebellum 7b R |
| ***CTRL < PMDD*** | | N.S. | | | | | |

All peak p-values are derived from Threshold-Free Cluster Enhancement (TFCE). Redundant peaks inside clusters are not listed. AAL, Automatic Anatomical Labelling atlas; FWE, Family Wise Error correction; PMDD, premenstrual dysphoric disorder; CTRL, healthy controls; L, left; R, right; N.S., non-significant.

**Table S7.** **Group comparison of grey matter volume over the whole brain, in the luteal phase of the menstrual cycle, after adjusting for psychiatric history.**

|  | **Cluster size** | | **p_FWE_** | **MNI coordinates** | | | **AAL region** |
| --- | --- | --- | --- | --- | --- | --- | --- |
|  |  |  |  | **x** | **y** | **z** |  |
| ***CTRL > PMDD*** | | | | | | | |
|  | 1593 | | 0.067 | -40 | -46 | -46 | Cerebellum 8 L |
|  |  | | 0.072 | -42 | -52 | -42 | Cerebellum Crus2 L |
|  |  | | 0.075 | -48 | -54 | -32 | Cerebellum Crus1 L |
|  |  | | 0.084 | -38 | -58 | -20 | Fusiform L |
|  |  | | 0.087 | -40 | -50 | -26 | Cerebellum 6 L |
|  | 529 | | 0.075 | 12 | -58 | -4 | Lingual R |
|  |  | | 0.088 | 0 | -60 | -6 | Vermis 4 5 |
|  |  | | 0.091 | 2 | -68 | -10 | Vermis 6 |
|  | 123 | | 0.084 | 12 | -86 | 8 | Calcarine R |
|  | 205 | | 0.093 | 20 | -78 | -12z | Lingual_R |
|  |  | | 0.098 | 6 | -86 | -3 | Calcarine R |
|  | 124 | | 0.094 | 42 | -51 | -46 | Cerebellum 7b R |
|  |  | | 0.099 | 38 | -54 | -54 | Cerebellum_8_R |
|  | 71 | | 0.095 | 15 | -82 | -28 | Cerebellum Crus1 R |
|  | 8 | | 0.098 | 26 | -8 | -30 | ParaHippocampal R |
|  | 17 | | 0.099 | 32 | -9 | -9 | Putamen R |
| ***CTRL < PMDD*** | | N.S. | | | | | |

All peak p-values are derived from Threshold-Free Cluster Enhancement (TFCE). Redundant peaks inside clusters are not listed. AAL, Automatic Anatomical Labelling atlas; FWE, Family Wise Error correction; PMDD, premenstrual dysphoric disorder; CTRL, healthy controls; L, left; R, right; N.S., non-significant.
